# A novel and accurate predictor of survival for patients with hepatocellular carcinoma after surgical resection: the neutrophil to lymphocyte ratio (NLR) combined with the aspartate aminotransferase/platelet count ratio index (APRI)

**DOI:** 10.1186/s12885-016-2189-1

**Published:** 2016-02-22

**Authors:** Fei Ji, Yao Liang, Shun-Jun Fu, Zhi-Yong Guo, Man Shu, Shun-Li Shen, Shao-Qiang Li, Bao-Gang Peng, Li-Jian Liang, Yun-Peng Hua

**Affiliations:** Organ Transplant Center, the First Affiliated Hospital, Sun Yat-sen University, Guangzhou, 510080 PR China; Department of Gastric and Pancreatic Surgery, Sun Yat-sen University Cancer Center, Guangzhou, 510060 PR China; Department of Pathology, the First Affiliated Hospital, Sun Yat-sen University, Guangzhou, 510080 PR China; Department of Liver Surgery, the First Affiliated Hospital, Sun Yat-sen University, Guangzhou, 510080 PR China

**Keywords:** Neutrophil to lymphocyte ratio, Aspartate aminotransferase/platelet count ratio index, Hepatocellular carcinoma, Prognosis, Biomarkers

## Abstract

**Background:**

The occurrence and development of hepatocellular carcinoma (HCC) depends largely on such non-tumor factors as inflammatory condition, immune state, viral infection and liver fibrosis. Various inflammation-based prognostic scores have been associated with survival in patients with HCC, such as the neutrophil/lymphocyte ratio (NLR), the platelet/lymphocyte ratio (PLR) and the prognostic nutritional index (PNI). The aspartate aminotransferase/platelet count ratio index (APRI) is thought to be a biomarker of liver fibrosis and cirrhosis. This study aims to evaluate the ability of these indices to predict survival in HCC patients after curative hepatectomy, and probe the increased prognostic accuracy of APRI combined with established inflammation-based prognostic scores.

**Methods:**

Data were collected retrospectively from 321 patients who underwent curative resection for HCC. Preoperative NLR, PLR, PNI, APRI and clinico-pathological variables were analyzed. Univariate and multivariate analyses were performed to identify the predictive value of the above factors for disease-free survival (DFS) and overall survival (OS).

**Results:**

Univariate analysis showed that NLR, PLR, PNI and APRI were significantly associated with DFS and OS in HCC patients with curative resection. Multivariate analysis showed that NLR and APRI were superior to PLR and PNI, and both were independently correlated with DFS and OS. Preoperative NLR >2 or APRI >1.68 predicted poor prognosis of patients with HCC after hepatectomy. Furthermore, the predictive range of NLR combined with APRI was more sensitive than that of either measure alone.

**Conclusions:**

Preoperative NLR and APRI are independent predictors of DFS and OS in patients with HCC after surgical resection. Higher levels of NLR or APRI predict poorer outcomes in HCC patients. Intriguingly, combining NLR and APRI increases the prognostic accuracy of testing.

## Background

Hepatocellular cancer (HCC) is one of the most common and most aggressive malignancies, the third leading cause of cancer-related deaths worldwide [1, 2]. Unlike other solid malignancies, most HCCs result from chronic liver disease [3], and the outcome of HCC depends in part on impaired liver function secondary to the above pathogenic condition, rather than solely to the tumor burden. Though Tumor Node Metastasis (TNM) staging system is an effective independent prognostic factor for HCC, its prognostic value is limited and lagging. A reliable prognostic index is therefore needed in routine clinical practice.

In addition to the intrinsic properties of cancer cells, host-related factors are increasingly recognized to influence the progression of tumors [4, 5]. For example, a systemic inflammatory response can impact tumor development through the inhibition of apoptosis, promotion of angiogenesis, and damage to the DNA. The pathogenesis of HCC is based on inflammation often caused by hepatotropic virus infection or ethanol consumption. Moreover, 70-90 % of HCCs are a result of cirrhosis [6, 7]. In recent studies, inflammation-based prognostic scores, such as the combination of albumin and lymphocyte counts used in the prognostic nutritional index (PNI) [5, 8], the combination of neutrophil and lymphocyte counts in the neutrophil/ lymphocyte ratio (NLR) [9–11], and the combination of platelet (PLT) and lymphocyte counts in the PLT/lymphocyte ratio (PLR) [12], have proved valuable in HCC prediction. In addition, recent studies [13–15] suggest that a simple and accurate biochemical marker of liver fibrosis and cirrhosis, i.e., the aspartate aminotransferase (AST)/ PLT count ratio index (APRI), may be 1) an indicator of postoperative prognosis in early stage hepatitis B (HBV)-related HCC patients, or 2) a marker of HCC risk in HBV patients,.

However, few studies have compared the prognostic value of these indices to predict tumor recurrence and survival after curative resection for HCC. Indeed, the combination of APRI and inflammation-based prognostic scores may increase the accuracy of prognosis prediction in patients who have undergone radical hepatectomy for HCC.

## Methods

### Study population

A total of 321 histologically proven HCC patients with hepatic resection from our hospital were recruited between 2006 and 2009. Written informed consent was obtained from all patients and this study complied with the standards of the Helsinki Declaration and current ethical guidelines and was approved by the Institutional Ethical Board of First Affiliated Hospital of Sun Yat-sen University. Routine assessment was performed within seven days before surgery, including a complete physical examination, hematologic and biochemistry profiles, chest X-ray, abdominal ultrasound and computed tomography (CT) or magnetic resonance imaging (MRI).

Eligibility criteria included: the International Union Against Cancer (seventh edition) TNM stage I, II, IIIA or IIIB [16]; Child-Pugh class A hepatic function; age 18-80 years; and written informed consent. Exclusion criteria included: TNM stage IIIC, IVA or IVB; existing second malignancy or history of second malignancy within the past five years; hematologic disorders; perioperative dysfunction of vital organs; or percutaneous ablation, transcatheter arterial chemoembolization (TACE), chemotherapy or radiotherapy within one month after surgery.

Blood samples were obtained before initial treatment to determine albumin, AST, alanine aminotransferase (ALT), total bilirubin (TBIL), white blood cell count, neutrophil count, lymphocyte count, platelet (PLT) count, prothrombin time and the a-fetoprotein (AFP) level. NLR, PLR, PNI and APRI were calculated using the following formulas:

$$ \mathsf{N}\mathsf{L}\mathsf{R}\kern0.5em =\kern0.5em \mathsf{Neutrophil}\ \mathsf{count}/\mathsf{lymphocyte}\ \mathsf{count}; $$

$$ \mathsf{P}\mathsf{L}\mathsf{R}\kern0.5em =\kern0.5em \mathsf{P}\mathsf{L}\mathsf{T}\ \mathsf{count}/\mathsf{lymphocyte}\ \mathsf{count}; $$

$$ \begin{array}{l}\mathsf{P}\mathsf{N}\mathsf{I}\kern0.5em =\kern0.5em \mathsf{10}\times \mathsf{serum}\ \mathsf{albumin}\ \left(\mathsf{g}/\mathsf{dl}\right) + \mathsf{0}.\mathsf{005}\kern0.5em \times \kern0.5em \mathsf{total}\ \mathsf{lymphocyte}\ \mathsf{count}\ \left(\mathsf{per}\ \mathsf{mm}\mathsf{3}\right);\ \\ {}\mathsf{and}\ \mathsf{APRI} = \left[\mathsf{A}\mathsf{S}\mathsf{T}\left(\mathsf{IU}/\mathsf{L}\right)/\mathsf{upper}\ \mathsf{limit}\ \mathsf{normal}\right]/\mathsf{P}\mathsf{L}\mathsf{T}\left(\times {\mathsf{10}}^{\mathsf{9}}/\mathsf{L}\right)\Big]\kern0.5em \times \kern0.5em \mathsf{10}\mathsf{0}.\end{array} $$

### Treatment and follow-up

Hepatectomy was defined as radical when there was no evidence of distant metastases and tumor clearance was complete both macroscopically and histologically. All patients were regularly followed up according to institutional practice, including liver ultrasound, chest X-ray and serum AFP every three months, and contrast CT every 6 months. Tumor relapse was defined by clinical, radiological and/or pathological diagnosis. After diagnosing recurrence, salvage treatments were selected, including re-operation, percutaneous ablation or TACE.

### Statistical analysis

Statistical analysis was performed using SPSS for Windows version 20.0 (SPSS, Chicago, IL, USA). Receiver operating characteristic (ROC) curve analysis was performed to select the most appropriate cut-off values for NLR, PLR, PNI and APRI to stratify patients at a high risk of death. The χ2 test was used to compare categorical variables. Disease-free survival (DFS) was calculated from the date of surgery to the date of recurrence, and overall survival (OS) from the date of surgery to the date of HCC-associated death. The Kaplan-Meier method was used to estimate the survival rates for different groups, and the equivalences of the survival curves were tested by log-rank statistics. The Cox proportional hazards model was used for univariate and multivariate survival analyses. *P* < 0.05 was considered statistically significant.

## Results

### Patient and tumor characteristics

The study included 285 male patients (88.8 %) and 36 female patients (11.2 %). The mean age was 51 years (range 21-79 years). A total of 235 patients (73.2 %) developed recurrence and 202 patients (62.9 %) died during follow up. Hepatitis B surface antigen (HBsAg) was positive in 281 patients (87.5 %) and cirrhosis in 253 (78.8 %) patients. Increased AFP levels (≥200 μg/L) were observed in 182 patients (56.7 %), and 95 patients (29.6 %) had multiple tumor masses. Mean tumor size was 87.6 mm (range 10-300 mm) in greatest diameter, and 210 (65.4 %) patients had tumors ≥ 50 mm in diameter. According to the Edmonson–Steiner stage of tumor differentiation, 248 (77.3 %) patients were in stages I–II and 73 (22.7 %) patients were in stages III–IV. Likewise, according to TNM classification, 185 patients were in TNM stage I and 137 patients in TNM stage II-III (Table 1).

**Table 1:** Prognostic factors for DFS and OS by univariate analysis

Variables	*n*	DFS			*P*	OS			*P*
		1-yr	3-yrs	5-yrs		1-yr	3-yrs	5-yrs	
Gender									
Male	285	43.5 %	29.8 %	25.8 %	0.096	69.5 %	44.6 %	37.1 %	0.026
Female	36	55.6 %	38.9 %	38.9 %		83.3 %	61.1 %	55.6 %	
Age(yrs)									
< 60	244	43.9 %	32.4 %	29.5 %	0.565	70.1 %	47.1 %	39.7 %	0.677
≥ 60	77	48.1 %	26.0 %	20.3 %		74.0 %	44.2 %	37.6 %	
HBsAg									
Positive	281	42.0 %	29.5 %	25.4 %	0.049	69.4 %	44.8 %	38.0 %	0.123
Negative	40	65.0 %	40.0 %	40.0 %		82.5 %	57.5 %	47.5 %	
AFP(μg/L)									
< 200	139	54.0 %	36.7 %	34.5 %	0.002	77.7 %	51.8 %	45.3 %	0.015
≥ 200	182	37.9 %	26.4 %	21.8 %		65.9 %	42.3 %	34.5 %	
ALT(U/L)									
< 80	265	47.5 %	32.4 %	28.5 %	0.108	73.2 %	49.8 %	41.8 %	0.019
≥ 80	56	32.1 %	23.2 %	21.4 %		60.7 %	30.4 %	26.8 %	
Hb(g/L)									
≤ 120	57	33.3 %	19.3 %	17.5 %	0.068	70.2 %	35.1 %	29.8 %	0.215
> 120	264	47.3 %	33.3 %	29.4 %		71.2 %	48.9 %	41.2 %	
WBC(×10^9^)									
< 10	287	46.0 %	31.7 %	27.8 %	0.384	72.1 %	46.3 %	39.6 %	0.795
≥ 10	34	35.3 %	23.5 %	23.5 %		61.8 %	47.1 %	35.3 %	
Lymphocyte(×10^9^)									
< 4	313	44.7 %	30.7 %	27.0 %	0.475	71.2 %	46.3 %	39.2 %	0.988
≥ 4	8	50.0 %	37.5 %	37.5 %		62.5 %	50.0 %	37.5 %	
TNM									
I	184	60.9 %	42.9 %	39.1 %	<0.001	84.8 %	61.4 %	55.4 %	<0.001
II-III	137	23.4 %	14.6 %	11.3 %		52.6 %	26.3 %	17.4 %	
Cirrhosis									
No	68	41.2 %	27.9 %	25.0 %	0.541	76.5 %	47.1 %	38.2 %	0.745
Yes	253	45.8 %	31.6 %	27.9 %		69.6 %	46.2 %	39.5 %	
PVTT									
No	263	52.5 %	35.7 %	31.8 %	<0.001	79.5 %	52.9 %	45.6 %	<0.001
Yes	58	10.3 %	8.6 %	6.9 %		32.8 %	17.2 %	10.1 %	
Tumor number									
single	226	54.0 %	38.5 %	34.0 %	<0.001	77.0 %	54.9 %	48.6 %	<0.001
multiple	95	23.2 %	12.5 %	11.0 %		56.8 %	26.3 %	16.7 %	
Tumor size(cm)									
< 5	111	69.4 %	51.4 %	43.9 %	<0.001	88.4 %	68.7 %	60.7 %	<0.001
≥ 5	210	31.9 %	20.0 %	18.5 %		61.4 %	34.3 %	27.5 %	
Complication									
No	273	44.7 %	32.6 %	28.4 %	0.356	72.2 %	46.9 %	40.6 %	0.345
Yes	48	45.8 %	20.8 %	20.8 %		64.6 %	43.8 %	31.0 %	
Tumor differentiation									
I-II	248	49.2 %	34.3 %	30.5 %	0.001	73.4 %	49.6 %	43.5 %	0.003
III-IV	73	30.1 %	19.2 %	16.4 %		63.0 %	35.6 %	24.5 %	
Resection margin(cm)									
< 2	184	42.1 %	26.8 %	23.4 %	0.138	69.9 %	41.5 %	34.3 %	0.041
≥ 2	137	48.2 %	35.7 %	32.0 %		72.3 %	52.6 %	45.2 %	
Intraoperative blood loss(ml)									
≤ 1000	247	77.3 %	51.8 %	44.9 %	<0.001	50.2 %	35.2 %	31.4 %	<0.001
> 1000	74	50.0 %	28.4 %	20.1 %		27.0 %	16.2 %	13.5 %	
NLR									
≤ 2	153	53.6 %	39.2 %	34.6 %	<0.001	79.7 %	58.8 %	49.6 %	<0.001
> 2	168	36.9 %	23.2 %	20.6 %		63.1 %	35.1 %	29.7 %	
PLR									
≤ 115	182	51.1 %	36.3 %	31.2 %	0.01	75.8 %	54.9 %	46.7 %	0.002
> 115	139	36.7 %	23.7 %	22.2 %		64.7 %	35.3 %	29.4 %	
APRI									
≤ 1.68	108	57.4 %	40.7 %	37.8 %	<0.001	83.3 %	59.3 %	51.8 %	<0.001
> 1.68	213	38.5 %	25.8 %	21.9 %		64.8 %	39.9 %	32.8 %	
PNI									
≤ 45	68	33.8 %	16.0 %	11.9 %	0.002	66.2 %	36.8 %	26.3 %	0.006
> 45	253	47.8 %	34.8 %	31.2 %		72.3 %	49.0 %	42.6 %	

*TNM* Tumor Node Metastasis, *AFP* Alpha-fetoprotein, *HBsAg* hepatitis B surface antigen, *PLT* platelet, *PVTT* portal vein tumor thrombi, *NLR* neutrophil/lymphocyte ratio, *PLR* platelet/lymphocyte ratio, *PNI* prognostic nutritional index, *APRI* Aspartate aminotransferase /platelet count ratio index

### Determination of cut-off value

**Fig. 1:**
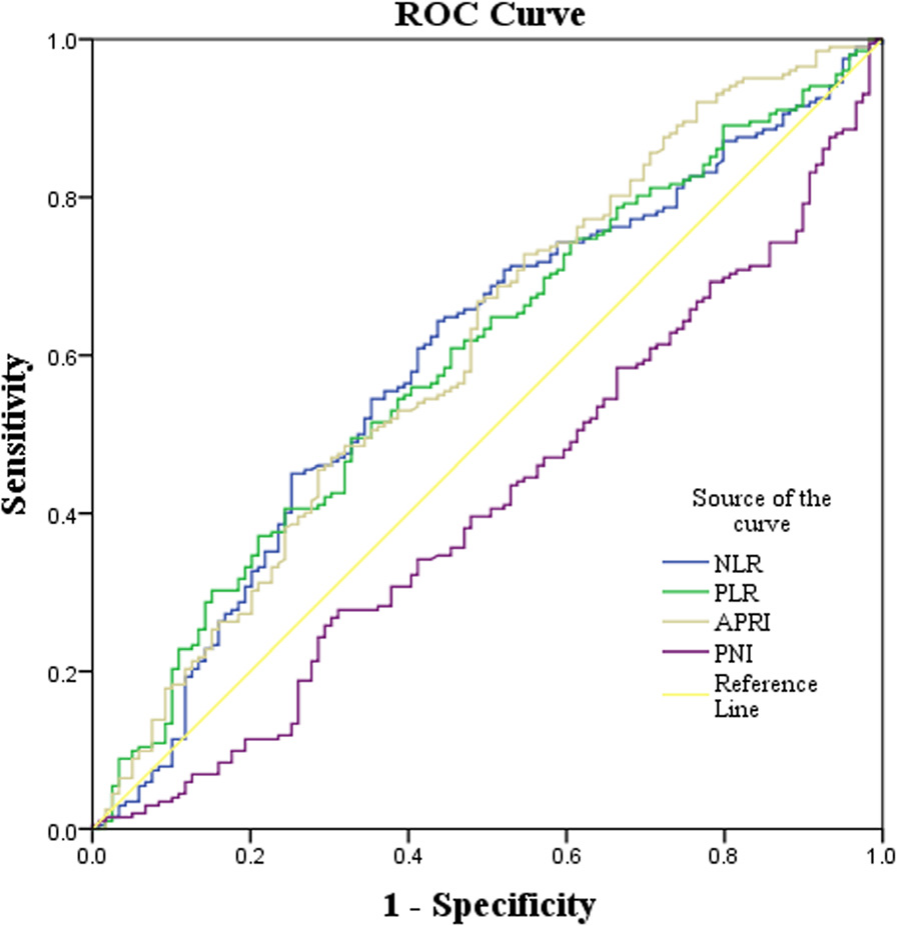
Determination of the cut-off value for NLR, PLR, PNI, APRI in HCC patients with hepatic resection

Using 5-year overall survival rate as an endpoint, stratification of each prognostic index was calculated by ROC curve analyses, according to the maximum joint sensitivity and specificity values based on the peak and cut-off points. Our results indicated that the optimal cut-off values for NLR, PLR, PNI and APRI were 2, 115, 45 and 1.68, respectively (Fig. 1).

### Independent prognostic factors for HCC

To further identify the risk factors linked to postoperative DFS and OS, NLR, PLR, PNI, APRI and 17 clinico-pathologic factors were evaluated by univariate analysis and the Cox regression model. Univariate analysis showed that the significant prognostic factors for DFS in HCC patients were HBsAg, AFP, TNM stage, tumor number, portal vein tumor thrombus (PVTT), tumor differentiation, intraoperative blood loss, NLR, PLR, PNI and APRI (all *P* < 0.05). Similarly, the significant factors for OS for those with HCC were gender, AFP, ALT, TNM stage, tumor number, PVTT, tumor differentiation, resection margin, intraoperative blood loss, NLR, PLR, PNI and APRI (all *P* < 0.05). After multivariate analysis, we found that AFP, TNM, PVTT, NLR and APRI were significant independent predictors of DFS (all *P* < 0.05), while TNM, PVTT, NLR, PLR and APRI were significant independent predictors of OS (all *P* < 0.05) (Tables 1 and 2).

**Table 2:** Independent prognostic factors for DFS and OS by the multivariate Cox proportional hazards regression model

Variables	DFS			OS		
	HR	95%CI	*P*	HR	95%CI	*P*
PLR				1.407	1.039-1.907	0.027
AFP	1.317	1.006-1.724	0.045			
TNM	1.981	1.469-2.672	<0.001	2.326	1.680-3.220	<0.001
PVTT	1.771	1.229-2.553	0.002	1.732	1.185-2.531	0.005
NLR	1.405	1.076-1.833	0.012	1.473	1.083-2.004	0.014
APRI	1.449	1.083-1.939	0.012	1.612	1.165-2.230	0.004

*HR* hazard ratio, *CI* confidence interval, *TNM* Tumor Node Metastasis, *AFP* Alpha-fetoprotein, *PVTT* portal vein tumor thrombi, *NLR* neutrophil/lymphocyte ratio, *PLR* platelet/lymphocyte ratio, *PNI* prognostic nutritional index, *APRI* Aspartate aminotransferase (AST)/platelet count ratio index

### Correlation between preoperative NLR or APRI and 17 clinico-pathologic characteristics in HCC

In order to further understand the prognostic value of preoperative NLR or APRI on HCC patients after resection, the relationships between NLR or APRI and 17 clinico-pathologic characteristics were analyzed. A NLR > 2 was more frequently observed in patients with high serum AFP levels (*P* = 0.015), with tumor size > 5 cm (*P* < 0 .001), those with PVTT (*P* = 0 .026), with recurrence (*P* = 0.005), with PNI ≤ 45 (*P* < 0.001) and with PLR > 115 (*P* < 0.001). Likewise, an APRI > 1.68 was associated with HBsAg (*P* = 0.015), a low PLT level (*P* < 0.001), a high ALT level (*P* < 0.001), TBIL (*P* = 0.003), cirrhosis (*P* = 0.001), PVTT (*P* < 0.001), recurrence (*P* = 0.001) and PNI ≤ 45 (*P* < 0.001) (Table 3).

**Table 3:** Correlation between preoperative NLR, APRI and clinico-pathologic characteristics in HCC

Variables	Cases	NLR		*P* value	APRI		*P* value
		≤2	>2		≤1.68	>1.68	
Age(yrs)							
≥ 60	77	32(41.6 %)	45(58.4 %)	0.219	24(31.2 %)	53(68.8 %)	0.598
< 60	244	121(49.6 %)	123(50.4 %)		84(34.4 %)	160(65.6 %)	
Gender							
Male	285	135(47.4 %)	150(52.6 %)	0.766	92(32.3 %)	193(67.7 %)	0.146
Female	36	18(50.0 %)	18(50.0 %)		16(44.4 %)	20(55.6 %)	
HCC family history							
Yes	24	8(33.3 %)	16(66.7 %)	0.144	8(33.3 %)	16(66.7 %)	0.973
No	297	145(48.8 %)	152(51.2 %)		100(33.7 %)	197(66.3 %)	
HBsAg							
Positive	281	134(47.7 %)	147(52.3 %)	0.982	86(30.6 %)	195(69.4 %)	0.002
Negative	40	19(47.5 %)	21(52.5 %)		22(55.0 %)	18(45.0 %)	
ALT(U/L)							
< 80	265	126(47.5 %)	139(52.5 %)	0.928	104(39.2 %)	161(60.8 %)	<0.001
≥ 80	56	27(48.2 %)	29(51.8 %)		4(7.1 %)	52(92.9 %)	
TBIL(μmol/L)							
< 34.2	294	144(49.0 %)	150(51.0 %)	0.119	106(36.1 %)	188(63.9 %)	0.003
≥ 34.2	27	9(33.3 %)	18(66.7 %)		2(7.4 %)	25(92.6 %)	
PLT(×10^9^)							
≥ 100	292	136(46.6 %)	156(53.4 %)	0.215	108(37.0 %)	184(63.0 %)	<0.001
< 100	29	17(58.6 %)	12(41.4 %)		0(0.0 %)	29(100.0 %)	
Cirrhosis							
Yes	253	126(49.8 %)	127(50.2 %)	0.139	74(29.2 %)	179(70.8 %)	0.001
No	68	27(39.7 %)	41(60.3 %)		34(50.0 %)	34(50.0 %)	
AFP(μg/L)							
≥ 200	182	76(41.8 %)	106(58.2 %)	0.015	57(31.3 %)	125(68.7 %)	0.313
< 200	139	77(55.4 %)	62(44.6 %)		51(36.7 %)	88(63.3 %)	
Tumor size(cm)							
> 5	210	76(36.2 %)	134(63.8 %)	<0.001	65(31.0 %)	145(69.0 %)	0.160
≤ 5	111	77(69.4 %)	34(30.6 %)		43(38.7 %)	68(61.3 %)	
Tumor number							
Single	226	112(49.6 %)	114(50.4 %)	0.295	80(35.4 %)	146(64.6 %)	0.305
Multiple	95	41(43.2 %)	54(56.8 %)		28(29.5 %)	67(70.5 %)	
TNM							
I	184	96(52.2 %)	88(47.8 %)	0.061	70(38.0 %)	114(62.0 %)	0.053
II-III	137	57(41.6 %)	80(58.4 %)		38(27.7 %)	99(72.3 %)	
Differentiation							
I-II	248	119(48.0 %)	129(52.0 %)	0.832	85(34.3 %)	163(65.7 %)	0.660
III-IV	73	34(46.6 %)	39(53.4 %)		23(31.5 %)	50(68.5 %)	
PVTT							
Yes	58	20(34.5 %)	38(65.5 %)	0.026	7(12.1 %)	51(87.9 %)	<0.001
No	263	133(50.6 %)	130(49.4 %)		101(38.4 %)	162(61.6 %)	
Recurrence							
Yes	235	101(43.0 %)	134(57.0 %)	0.005	67(28.5 %)	168(71.5 %)	0.001
No	86	52(60.5 %)	34(39.5 %)		41(47.7 %)	45(52.3 %)	
PNI							
≤ 45	68	15(22.1 %)	53(77.9 %)	<0.001	10(14.7 %)	58(85.3 %)	<0.001
> 45	253	138(54.5 %)	115(45.5 %)		98(38.7 %)	155(61.3 %)	
PLR							
≤ 115	182	118(64.8 %)	64(35.2 %)	<0.001	55(30.2 %)	127(69.8 %)	0.137
> 115	139	35(25.2 %)	104(74.8 %)		53(38.1 %)	86(61.9 %)	
Complication							
No	273	130(47.6 %)	143(52.4 %)	0.970	92(33.7 %)	181(66.3 %)	0.960
Yes	48	23(47.9 %)	25 (52.1 %)		16(33.3 %)	32(66.7 %)	

### Overall and disease free survival rates according to NLR or APRI

**Fig. 2:**
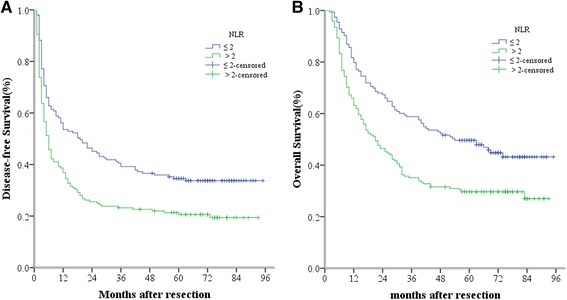
Relationship between NLR and DFS/OS of HCC patients after hepatectomy. **a** DFS of patients with NLR > 2 was significantly shorter than those with NLR ≤ 2 (*P* < 0.001, log-rank test). **b** OS of patients with NLR > 2 was also markedly shorter than those with NLR ≤ 2 (*P* < 0.001, log-rank test)

To determine the ability of NLR and APRI to predict OS and DFS, the 321 HCC patients were divided into two groups according to their NLR profiles: the NLR ≤ 2 group (n = 153) and the NLR > 2 group (n = 168). Using the Kaplan-Meier method to analyze patient survival, we found that the 1-, 3- and 5-year DFS rates of the NLR ≤ 2 group were markedly higher than those of the NLR > 2 group (53.6 %, 39.2 % and 34.6 % vs 36.9 %, 23.2 % and 20.6 %, respectively, *P* < 0.001) (Fig. 2a), while the 1-, 3- and 5-year OS rates of the NLR ≤ 2 group were also significantly higher than those of the NLR > 2 group (79.7 %, 58.8 % and 49.6 % vs 63.1 %, 35.1 % and 29.7 %, respectively, *P* < 0.001) (Fig. 2b). Our findings therefore indicated that high NLR levels were correlated with a low survival rate in patients with HCC.

**Fig. 3:**
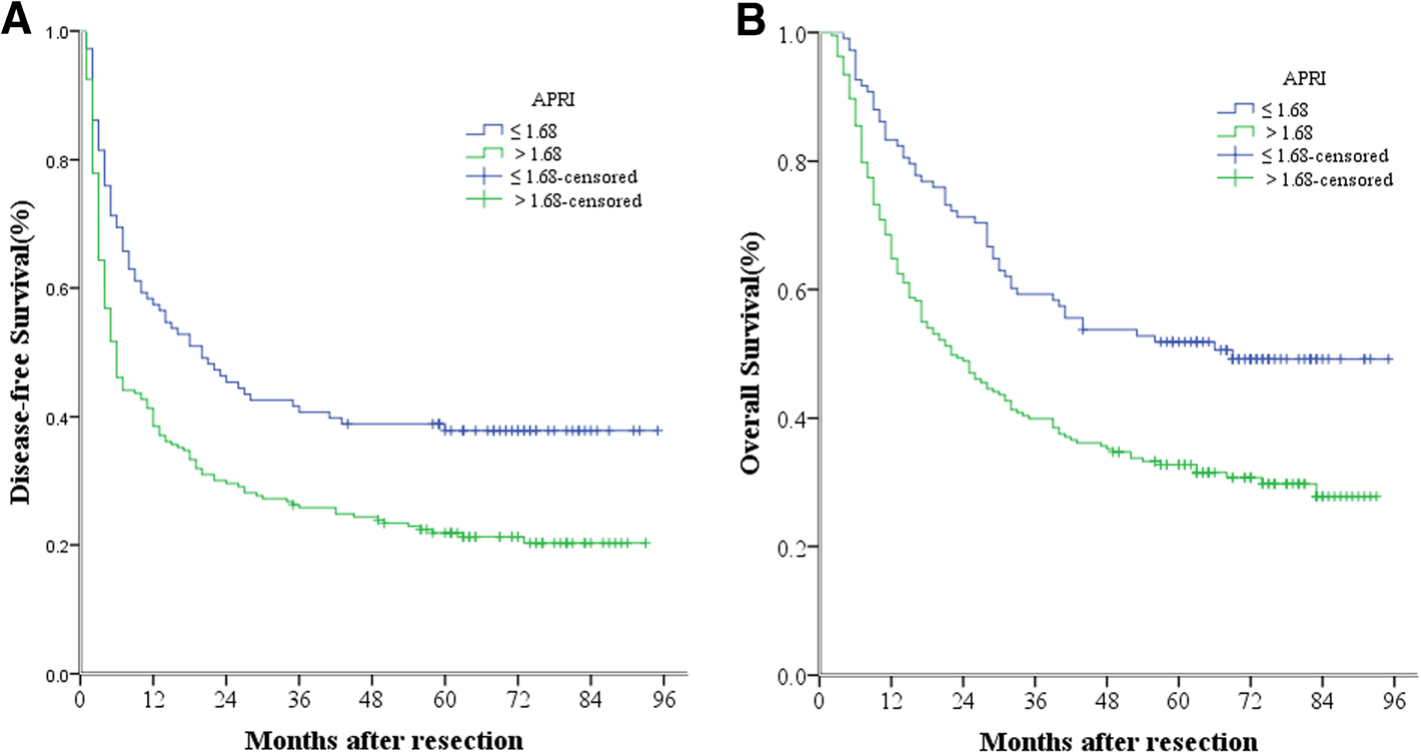
Relationship between APRI and DFS/OS of HCC patients after hepatectomy. **a** DFS of patients with APRI > 1.68 was significantly shorter than those with APRI ≤ 1.68 ( *P* < 0.001, log-rank test). **b** OS of patients with APRI > 1.68 was also markedly shorter than those with APRI ≤ 1.68 ( *P* < 0.001, log-rank test)

Likewise, the HCC patients were divided into two groups according to their APRI profiles: the APRI ≤ 1.68 group (n = 108) and the APRI > 1.68 group (n = 213). The 1-, 3- and 5-year DFS rates of the APRI ≤ 1.68 group were markedly higher than those of the APRI > 1.68 group (57.4 %, 40.7 % and 37.8 % vs 38.5 %, 25.8 % and 21.9 %, respectively, *P* < 0.001) (Fig. 3a). Also, the 1-, 3- and 5-year OS rates of the APRI ≤ 1.68 group were significantly higher than those of the APRI > 1.68 group (83.3 %, 59.3 % and 51.8 % vs 64.8 %, 39.9 % and 32.8 %, respectively, *P* < 0.001) (Fig. 3b). A high APRI level therefore implied poor DFS and OS in HCC patients with hepatectomy.

### The prognostic value of NLR combined with APRI for HCC after hepatectomy

**Fig. 4:**
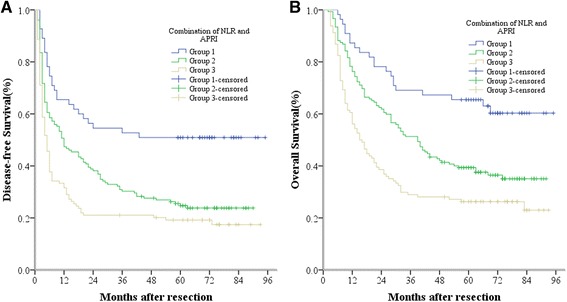
The combination of NLR and APRI was found to enhance prognostic accuracy for HCC. Disease-free survival curves (panel **a**) and overall survival curves (panel **b**). Group1, both NLR ≤ 2 and APRI ≤ 1.68; Group2, both NLR > 2 and APRI ≤ 1.68 or both NLR ≤ 2 and APRI > 1.68; Group3, both NLR > 2 and APRI > 1.68

To analyze the prognostic value for HCC survival of combining NLR and APRI, we set up NLR ≤ 2 or APRI ≤ 1.68 as the score of 0 and NLR > 2 or APRI > 1.68 as the score of 1. After combining NLR with APRI, patients were allocated into three groups. Patients with both NLR ≤ 2 and APRI ≤ 1.68 were calculated to have a score of 0, and were recruited into group 1. Patients with NLR > 2 and APRI ≤ 1.68 or with NLR ≤ 2 and APRI > 1.68 were in Group 2, with the total score of 1. Finally, those with both NLR > 2 and APRI > 1.68 were in Group 3 with the total score of 2 (Table 4). Patients in Group 1 had the best DFS and OS rates, followed by those in Group 2; patients in Group 3 had the worst prognosis. The 1-, 3- and 5-year DFS rates of Group 1 (65.5 %, 52.7 % and 50.9 %, respectively) were significantly higher than those of Group 2 (47.4 %, 30.3 % and 24.7 %, respectively, *P* = 0.001) and Group 3 (31.6 %, 21.1 % and 19.2 %, respectively, *P* < 0.001). Similarly, the 1-, 3- and 5-year OS rates of Group 1 (87.3 %, 69.1 % and 65.5 %, respectively) were also significantly higher than those of Group 2 (76.3 %, 51.3 % and 39.3 %, respectively, *P* = 0.002) and Group 3 (56.1 %, 28.9 % and 26.3 %, respectively, *P* < 0.001) (Fig. 4a and b). Furthermore, we found that the 1-, 3- and 5-year DFS and OS rates of Group 2 were both significantly higher than those of Group 3 (*P* = 0.013 and *P* = 0.002).

**Table 4:** A novel and accurate predictor for HCC: the combination of NLR and APRI

Variable	Score
NLR	
≤ 2	0
> 2	1
APRI	
≤ 1.68	0
> 1.68	1
Prognostic stratification	
0	Low risk of mortality
1	Intermediate risk of mortality
2	High risk of mortality

## Discussion

Surgical resection is the mainstay of curative therapy for HCC. However, it is well understood that malnutrition is of particular concern in patients with HCC due to the concomitant underlying fibrosis and cirrhosis [17]. In addition, hepatectomy can cause a series of postoperative changes in the metabolic, endocrine, neuroendocrine and immune systems, which can impair immunological functions and contribute to an increased risk of postoperative complications and metastasis in HCC patients after surgery. Theoretically, malnutrition, immunological status, fibrosis and cirrhosis may act as predictors of survival in HCC patients after curative resection.

Increasing evidence seems to indicate that the host inflammatory response is correlated with the occurrence and development of HCC, so that it can serve to predict the clinical outcomes of patients with HCC. Several studies have shown that an elevated NLR is associated with poor prognosis in patients with HCC undergoing surgical resection [18], transplantation [19], transarterial chemoembolisation [20] and radiofrequency ablation [9]. PNI was first reported by Onodera et al. in Japan for the assessment of the immunologic and nutritional status of patients [21]. Pinato et al. [5] first demonstrated that PNI was useful for assessing prognosis in patients with HCC. Chan et al. [8] indicated that PNI was a significant prognostic factor for OS and DFS of patients with very early/early stage HCC receiving curative surgery. Okamura and colleagues [22] further pointed out that NLR and PNI were predictors of overall survival in patients who had undergone hepatectomy for HCC with curative intent. PLR is proposed to be a predictor of thrombotic and inflammatory conditions, thus making it an attractive inflammation-related biomarker for tumors also. For HCC, Xue et al. [23] indicated that a high baseline PLR is a useful predictor of poor survival in patients undergoing chemoembolization. Xia et al. [24] also found that a high level of pre-transplant PLR was associated with advanced tumor stage and aggressive tumor behavior, making it a useful indicator for post-transplant HCC recurrence.

In our study, we compared the prognostic value of various inflammation markers, based on prognostic scores for NLR, PLR and PNI. We found that preoperative NLR, PLR and PNI were all significant prognostic factors for DFS and OS in patients with HCC. High levels of NLR (>2), high levels of PLR (>115) and low levels of PNI (≤45) predicted poor prognosis in HCC patients with curative resection. However, NLR was superior in this aspect to PLR and PNI; what is more, NLR was the only independent predictive factor for both DFS and OS in HCC patients. In addition, PLR was the only independent predictive factor for OS in HCC patients. We also found a significant correlation between NLR and several clinico-pathological characteristics: AFP, PVTT, tumor size, tumor encapsulation, recurrence, PNI and PLR.

A three-step process of hepatitis → liver fibrosis/cirrhosis → HCC is believed to be the primary mechanism of hepatocarcinogenesis. Wu and colleagues [25] reported that high viral loads and hepatic inflammatory activity were associated with late HCC recurrence and that tumor factors were associated with early HCC recurrence. Hung et al. [26] found that the degree of liver fibrosis is associated with tumor recurrence as well as with overall survival in small and solitary HBV-related HCC patients with surgical resection. Although liver biopsy is the gold standard for the assessment of liver fibrosis, it is a high-risk procedure and usually complicated by pain, bleeding, hemothorax, bile duct injury or risk of penetration of the abdominal viscera. In addition, sampling errors and inter-observer variation decrease the reliability of liver biopsy as a predictive tool. Recently, APRI was validated as a simple, feasible, noninvasive way to assess the degree of liver fibrosis in patients with chronic hepatitis B or C [27, 28]. APRI was also found to be a prognostic biomarker in small HCC patients after radiofrequency ablation therapy and surgical resection [14, 29]. APRI was also found useful in predicting HCC risk in patients with nonalcoholic fatty liver disease [30] or HBV.

Our results showed that APRI was superior to PLR or PNI as an independent predictor of both DFS and OS in HCC patients with hepatectomy. APRI > 1.68 predicted a short DFS and OS in HCC patients. In correlation analysis, we found that, as in previous studies [27, 28], APRI was intimately associated with cirrhosis and HBV status. High APRI levels predicted the condition of cirrhosis and HBV infection. In addition, high APRI levels were positively correlated to PVTT, incomplete tumor encapsulation and tumor relapse, and negatively correlated to PNI.

As mentioned above, the non-tumor factors, including inflammatory condition, immune state, viral infection and liver fibrosis, play an important role in determining tumor recurrence in HCC patients. Accordingly, NLR and APRI were both independent predictors of both DFS and OS in HCC patients. Given that NLR is an accurate inflammatory marker, and APRI can assess the degree of liver fibrosis, we hypothesize that the combination of NLR and APRI reflects the range of non-tumor factors, thereby enabling more precise prediction of outcomes in HCC patients with curative resection.

Excitingly, our results showed that the combination of NRL and APRI has a better prognostic value than either one alone. For example, the data showed that patients in Group 1 (both NLR ≤ 2 and APRI ≤ 1.68) had the best DFS and OS rates, with 1-, 3- and 5-year DFS rates of 65.5 %, 52.7 % and 50.9 %, respectively, and 1-, 3- and 5-year OS rates of 87.3 %, 69.1 % and 65.5 %, respectively. However, the prognosis was worst in patients in Group 3 (both NLR > 2 and APRI > 1.68), whose 1-, 3- and 5-year DFS rates were only 31.6 %, 21.1 % and 19.2 %, respectively, and whose 1-, 3- and 5-year OS rates were only 56.1 %, 28.9 % and 26.3 %, respectively. The outcomes for patients in Group 2 (both NLR > 2 and APRI ≤ 1.68, or both NLR ≤ 2 and APRI > 1.68), fell between those of the above two groups, with 1-, 3- and 5-year DFS rates of 47.4 %, 30.3 % and 24.7 %, respectively, and 1-, 3- and 5-year OS rates of 76.3 %, 51.3 % and 39.3 %, respectively. Furthermore, the difference between any two groups was significant. So the combination of NLR and APRI inevitably possesses both accurate and clinically meaningful prognostic value for HCC patients with curative resection, for example, both NLR > 2 and APRI > 1.68 represents high risk of mortality, both NLR ≤ 2 and APRI ≤ 1.68 represents low risk of mortality, and the other represents intermediate risk of mortality. It is very easy and valuable to accurately predict the prognosis of HCC patients in clinical. Its mechanisms may be that the combination of NLR and APRI reflects most of non-tumor factors, including inflammatory condition, immune state, viral infection and liver fibrosis, rather than a certain aspect of non-tumor factors. So we can speculate that the combination of NLR and APRI is useful to guide the follow-up and further treatment of HCC patients after curative resection. For example, we should follow up closely so as to find the early tumor recurrence for the patients with both NLR > 2 and APRI > 1.68. It may be better to follow up once a month for this kind of patients. Even we can give some initiative treatments to these patients, such as TACE, systemic chemotherapy, or cellular immunotherapy, etc.

Certainly, the present study has some limitations. First, it is a retrospective, single- institution study with a relatively small number of patients. A well-designed, prospective study with a larger number of patients with HCC who underwent radical surgery is needed. Second, we were not able to split our data set into a training data set and a test data set for statistical validation because of the small number of patients. So it is worthwhile to conduct additional studies to validate our findings and test their clinical applicability in HCC treatment in the future.

## Conclusions

In summary, our study determined that NLR and APRI were superior to PNI and PLR, and were independent predictors of the prognosis for HCC patients with curative resection. Their high levels predicted poor outcomes in HCC patients: early recurrence and short lifetime. Excitingly, NLR combined with APRI was more sensitive than either scale alone in predicting DFS and OS in patients with HCC. The combination of NLR and APRI may be a useful prognostic tool to determine survival in patients with HCC after resection, and to further guide their follow-up and postoperative treatment.

## Abbreviations

AFP: alpha-fetoprotein
ALT: alanine aminotransferase
APRI: aspartate aminotransferase/platelet count ratio index
AST: aspartate aminotransferase
CT: computed tomography
DFS: disease-free survival
HBsAg: hepatitis B surface antigen
HBV: Hepatitis B
HCC: hepatocellular cancer
MRI: Magnetic resonance imaging
NLR: neutrophil/lymphocyte ratio
OS: overall survival
PLR: platelet/lymphocyte ratio
PLT: platelet
PNI: prognostic nutritional index
PVTT: portal vein tumor thrombus
ROC: receiver operating characteristics
TACE: transcatheter arterial chemoembolization
TBIL: total bilirubin.
TNM: Tumor Node Metastasis
